# Clinical evaluation of a novel disposable neurostimulator used to accelerate regeneration of injured peripheral nerves in the hand

**DOI:** 10.1186/s42234-025-00171-y

**Published:** 2025-04-25

**Authors:** Christopher J Coroneos, Carolyn Levis, Michael P Willand, Katelyn JW So, James R Bain

**Affiliations:** 1 Hamilton Health Sciences, 237 Barton Street East, Hamilton, Ontario L8L 2X2 Canada; 2 Division of Plastic Surgery, St. Joseph’s Healthcare Hamilton, 50 Charlton Avenue, Hamilton, Ontario L8S 4K1 Canada; 3 Division of Plastic Surgery, McMaster University, 1200 Main St W, Hamilton, Ontario L8N 3Z5 Canada; 4 Epineuron Technologies Inc., 1875 Buckhorn Gate Suite 602, Mississauga, Ontario L4W 5P1 Canada

**Keywords:** Peripheral nerve regeneration, Nerve repair, Perioperative electrical stimulation, Nerve surgery

## Abstract

**Background:**

Preclinical and early clinical evidence demonstrates that electrical stimulation (ES) applied for one hour following surgical nerve intervention enhances axonal regeneration and functional outcomes. Wide clinical implementation however, has been hindered by a lack of suitably designed stimulators. The aim of this pilot study was to investigate sensory recovery, safety, tolerability, and RCT feasibility for the use of a novel single-use stimulator to deliver ES therapy in an acute nerve transection cohort.

**Methods:**

Patients with complete transection of a proper digital nerve were included in the trial. An investigational version of PeriPulse^TM^ was used with intraoperative electrode implantation and 1-hour ES therapy delivered postoperatively. Patient tolerance was assessed during stimulation and visual-analogue pain scores were collected at the first post-operative visit. At 3- and 6-months post-op, sensory recovery and quality of life were assessed using 2-point discrimination, monofilament tests, and the Disability of Arm, Shoulder, and Hand (DASH) questionnaire, respectively.

**Results:**

A total of 10 patients were enrolled. Intraoperative electrode placement did not impact operating room time, taking less than 5 minutes to implement. There were no related adverse events. Participants reported tolerable stimulation during ES therapy with no reports of pain. At the first post-operative visit patients had a mean visual-analogue pain score of 0.6 (range 0 - 1.9). Pressure threshold detection significantly improved between baseline, 3 months and 6 months. A greater proportion of ES treated patients (87.5%) had improved hand pressure thresholds (diminished light touch or diminished protective sensation) at 6 months compared to a historical comparator group. DASH scores improved over the timeline. Participants treated with ES therapy experienced minimal postoperative functional disability.

**Conclusions:**

The use of the PeriPulse^TM^ prototype for the delivery of perioperative ES therapy was safe, well-tolerated, and usable. Sensory recovery was demonstrated and a larger RCT is feasible.

**Trial Registration:**

NCT04732936; 2021 - 01 - 29

## Background

Peripheral nerve injuries are present in at least 2.5% of trauma cases in the United States, annually (Padovano et al. 2022). They represent a substantial burden on the health care system, with at least $150B in annual costs (Grinsell and Keating 2014; Taylor et al. 2008). While injured peripheral nerves have the capacity for regeneration (Gordon 2024), the process is slow and recovery is not guaranteed or predictable due to the inefficiency and barriers to the regeneration process (e.g., staggered and misdirected regeneration (Lundborg 2005; Sulaiman and Gordon 2013; Gordon et al. 2003; Brushart et al. 2002; Gordon et al. 2015) and a hostile microenvironment (Gordon et al. 2015; Burnett and Zager 2004; Al-Majed et al. 2000). While there have been numerous advances in therapies to enhance outcomes following nerve injuries and repairs, the vast majority of investigations have not been translated into the clinical environment.

**Fig. 1:**
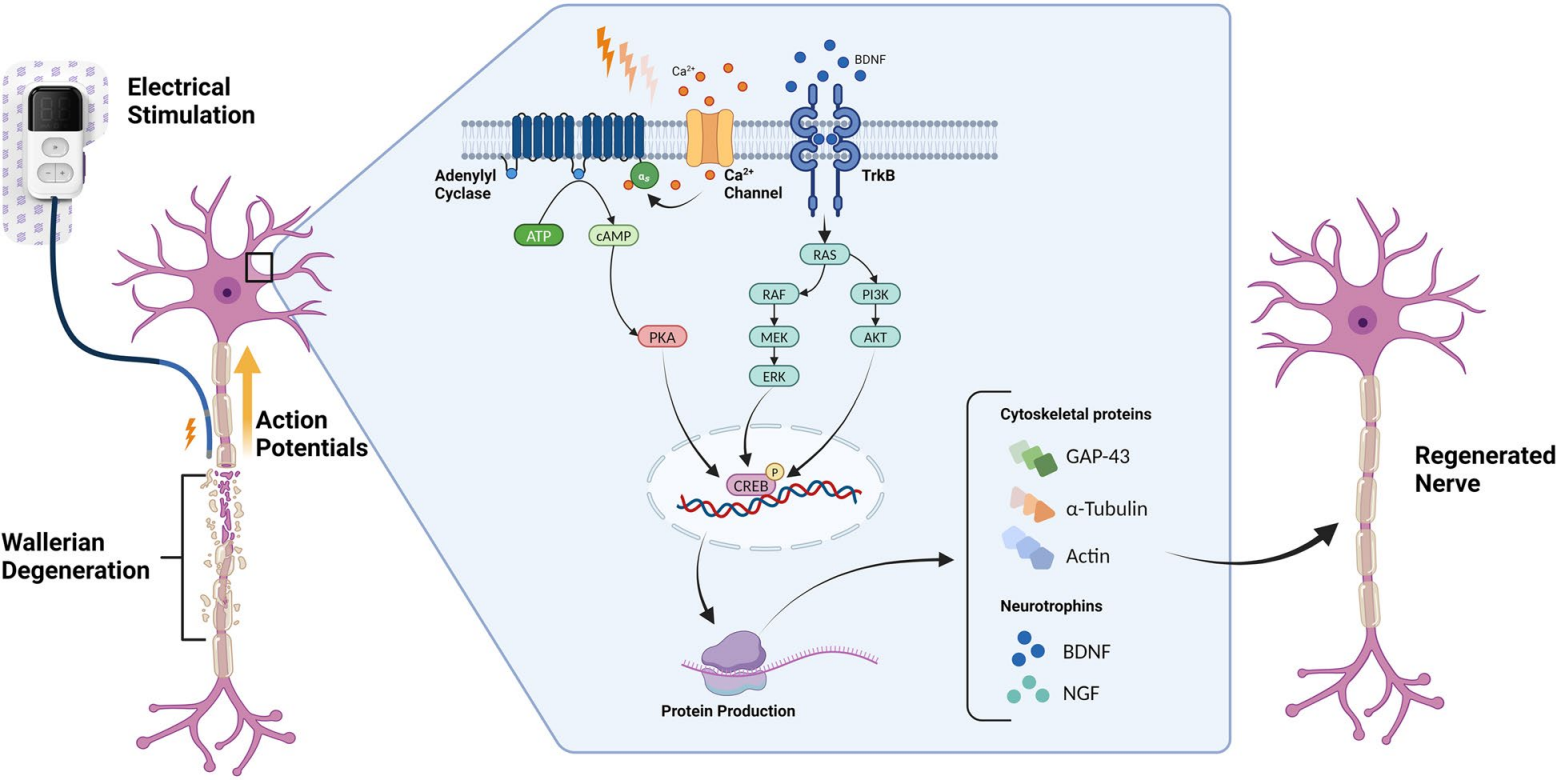
The molecular mechanism of electrical stimulation is largely driven by upregulation of BDNF through the conduction of action potentials to the cell body. Numerous pathways are activated that result in increased production of proteins that are used to facilitate axon regeneration. Created in BioRender. Willand, M. (2024) BioRender.com/r99z234

Electrical stimulation (ES) of injured peripheral nerves has long been shown in pre-clinical animal models to promote regeneration of both motor and sensory nerves (Ahlborn et al. 2007; English et al. 2007; Hetzler et al. 2008; Lal et al. 2008; Vivó et al. 2008; Asensio-Pinilla et al. 2009; Sharma et al. 2009; Yeh et al. 2010; Foecking et al. 2012; Singh et al. 2012; Zuo et al. 2020). Gordon and Brushart discovered a time titration effect in the application of ES and in a seminal paper published by their group, described how prolonged ES (24 hours per day for 1 or 2 weeks)can be reduced to a single, 1-hour session (Al-Majed et al. 2000). The mechanism of action is mediated through action potential conduction towards the neuronal soma with sodium channel blockers abolishing the benefit of ES when applied proximal to the ES delivery site (Al-Majed et al. 2000). The requirement of action potential conduction was further confirmed through the application of optical stimulation of CH2R expressing axons. In this experiment, only the axons expressing CH2R were able to be depolarized thus eliminating any supporting cells from being activated (Park et al. 2015; Ward et al. 2016; Ward et al. 2018). The current understanding of the molecular mechanisms involved center around Ca^2+^ mediated cyclic adenosine monophosphate (cAMP) elevation. This drives the upregulation of neurotrophins and their receptors such as brain derived neurotrophic factor (BDNF), neurotrophin 4/5 (NT- 4/5), and nerve growth factor (NGF), tropomyosin receptor kinase A and B (trkA, trkB), and p75. The upregulation of neurotrophins amplifies the cAMP response. The over expression of cAMP is important as this causes the expression of several axon growth related proteins, for example, actin, tubulin, and growth associated protein- 43 (GAP- 43) (Gordon 2024; Senger et al. 2024; Javeed et al. 2021). Figure 1 illustrates this mechanism.

ES also impacts the supporting cells such as macrophages and Schwann cells located in the distal nerve stump. ES delivered for a single hour was shown to change the phenotype of macrophages from a pro-inflammatory (M1) type to a pro-repair type (M2) macrophage (McLean and Verge 2016). Schwann cells have also been impacted in a similar manner with ES inducing a repair phenotype and allowing for faster Wallerian degeneration.

Early translational research using off-the-shelf neuroscience-type stimulators (Grass SD9) has demonstrated preliminary safety and efficacy of the single hour dose of stimulation. These randomized clinical studies investigated the use of ES following various types of nerve surgeries such as: nerve decompression surgery at the wrist or elbow (carpal or cubital tunnel syndrome), direct nerve repair (digital nerve laceration), or partial nerve devascularization occurring during neck dissection. In all cases the results demonstrated that ES provided patients with benefits ranging from an increase in the number of motor units (more functional connections at the muscle), improved grip strength, to increased tactile discrimination and sensation. Taken together, brief 1-hour, direct electrical stimulation of injured nerves has emerged as a frontrunner therapy for adoption, demonstrated by the robust pre-clinical literature supporting efficacy in small animal models (Brushart et al. 2002; Al-Majed et al. 2000; Ahlborn et al. 2007; Vivó et al. 2008; Asensio-Pinilla et al. 2009; Singh et al. 2012; Park et al. 2015; Ward et al. 2016; Al-Majed et al. 2000; Geremia et al. 2007; Brushart et al. 2005; Wang et al. 2009; Franz et al. 2013; Haastert-Talini and Grothe 2013; Elzinga et al. 2015) and early clinical efficacy in randomized controlled trials (Barber et al. 2018; Gordon et al. 2010; Wong et al. 2015; Power et al. 2019). Despite this abundance of research, wide adoption of this therapy has not occurred. This may be due to the lack of systematic research aimed at clinical applications and the fact that most of the current research focuses on animal models or laboratory environments. The literature has often cited that the implementation is limited to intra-operative use and that longer operative times are undesirable and cost-prohibitive (Juckett et al. 2022; Roh et al. 2022). Furthermore, there are no devices specifically designed to implement this short duration therapy resulting in use of laboratory equipment and custom fabricated or alternate use electrodes that are not designed for this purpose or are outdated (Gordon et al. 2010). Others have attempted to use hand-held stimulators in an off-label application to deliver shorter duration (10 minutes) stimulation in order to fit within an existing surgical procedure (Roh et al. 2022; Evans et al. 2021; Saffari et al. 2024). The latter was accomplished by holding the stimulator’s probe on the nerve for 10 minutes while monitoring the elapsed time. While data is limited on the shorter duration approach, clinicians are still faced with the problem of using a device that is not specifically designed to deliver this therapy in a reliable and repeatable manner. A novel, single-use stimulator was developed to address the limitations of existing hardware and minimize surgical operating time while still delivering the well studied 1-hour stimulation paradigm. Specifically, the single-use stimulator addressed these concerns by having pre-programmed stimulation frequency and duration within the battery powered stimulator, an implantable electrode designed to conform to the nerve, anode and cathode placement to deliver precise stimulus to the nerve, and a smooth surface not requiring fixation allowing safe, comfortable removal after closure of the surgical wound. In this paper we present clinical safety and tolerability in a digital nerve transection model using this novel stimulator. Nerve recovery was evaluated 3- and 6-months post nerve repair using validated sensory outcome measures. This device was designed to deliver brief electrical stimulation in a peri-operative setting, allowing intraoperative electrode placement and delivery of postoperative therapeutic stimulation.

## Methods

This was an open-label feasibility multi-center clinical trial. The study was conducted in compliance with the Declaration of Helsinki and approved by the Hamilton Integrated Research Ethics Board. The trial is registered with ClinicalTrials.gov (NCT04732936).

### Participants

Patients with complete and single digital nerve transections were recruited from hand clinics or the emergency department at Hamilton Health Sciences Centre and St. Joseph’s Healthcare Hamilton (Ontario, Canada). Informed consent was obtained prior to enrollment. Participants were limited to consenting male and female adults from 18–65 years of age. Participants must have had a completely lacerated proper digital nerve (common branch lacerations were excluded, as well as injuries in zone 1) and be operated on within 14 days of sustaining their injury. Concomitant bone injury in the same digit was excluded as this may require prolonged immobilization and may confound the regeneration and assessment of the sensory nerve being studied. Investigational devices were not used on any patients with implanted devices (i.e., cardiac pacemakers, defibrillators, or metallic implants). Patients with notable comorbidities, allergies, and medications were not enrolled in the study.

### Preoperative evaluation

Standard of care preoperative evaluations were conducted. Participants with a suspected digital nerve injury were assessed by a qualified hand therapist. Baseline sensory function was assessed on the affected digit via Semmes-Weinstein Monofilament Test (SWMT) and Static 2-Point Discrimination (S2PD). Baseline functional disability was determined by the use of the Disability of the Arm, Shoulder, and Hand (DASH) questionnaire.

### Surgical procedure and investigational PeriPulse^TM^ implementation

Standard of care procedures were conducted by qualified plastic and reconstructive surgeons under either general or local anesthesia (Xylocaine 1%, no epinephrine). Regional and/or long-acting local anaesthesia were not used. Digital nerves were trimmed and directly repaired using two or three 8-0 or 9-0 nylon epineurial sutures. Additional coaptation aids such as fibrin glue or nerve wraps were not used. Following the surgical intervention, an investigational version of PeriPulse^TM^, a single-use and battery-powered temporary peripheral nerve stimulation system manufactured by Epineuron Technologies Inc., was implemented in the operating room. This kit contained the signal generator, a multi-contact shapeable electrode lead, a surface return electrode, and an introducer tool. The multi-contact lead and surface electrode allowed for physician selection of monopolar or bipolar electrode configurations which was used to determine patient comfort and ease of implementation. Selection of one configuration over the other was purely subjective and driven by usability of the device. Bipolar configurations may lead to higher current densities and a more targeted stimulation of the nerve whereas monopolar configurations create more disperse electrical fields that may stimulate other structures near the treated nerve.

**Fig. 2:**
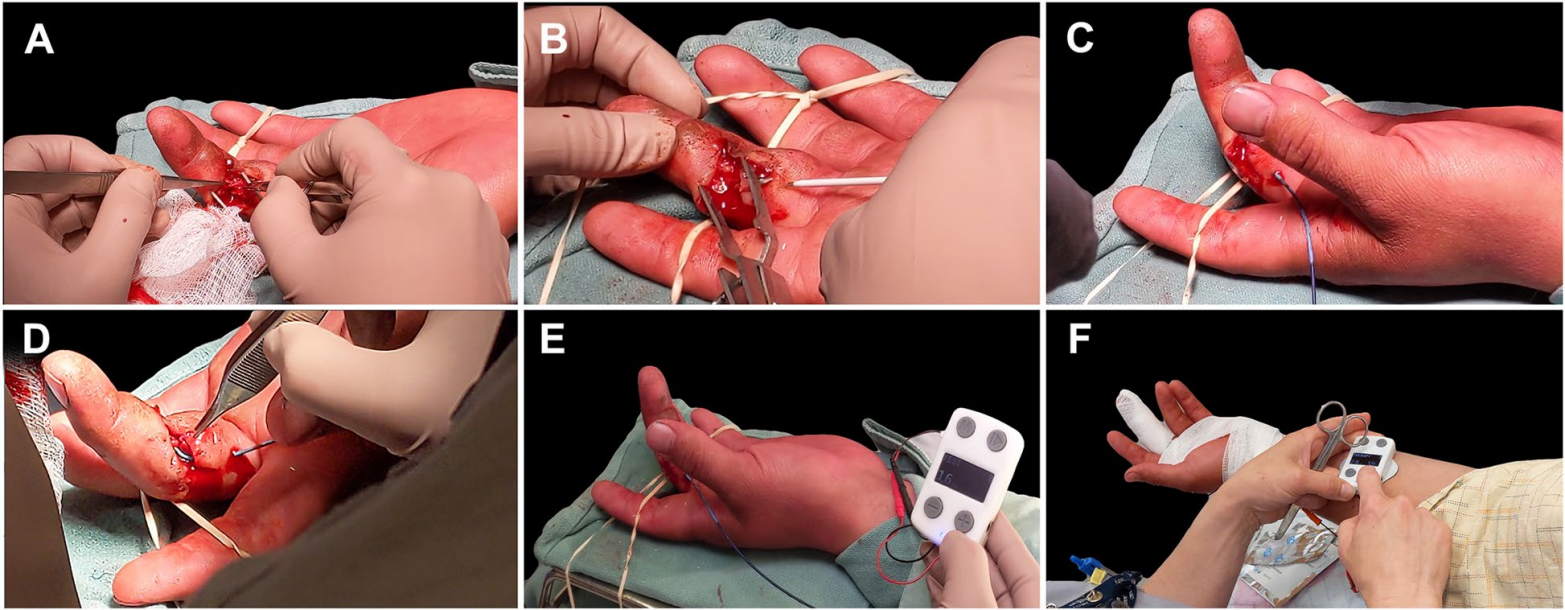
Surgical implementation of Investigational PeriPulse^TM^ to enable perioperative ES therapy in a digital nerve transection and repair model under either general anesthesia or short-acting local anesthesia. Image background for each panel was digitally removed. **A** Nerve repair occurs as per standard of care. **B** Introducer tool is used to create a para-incisional access point for the electrode lead. **C** Electrode lead is inserted through the introducer tool and placed proximal to the nerve coaptation under visual guidance. The introducer tool is removed. **D** The electrode lead may be shaped according to the surrounding anatomy to secure placement and prevent shifting of the contacts. Wound closure proceeds as per standard of care. **E** Signal generator is connected to the electrode lead. Stimulation intensity (mA) is adjusted to the patient’s tolerable range. **F** 1-hour ES therapy is initiated while the hand is dressed and continues as the patient is transferred to the recovery area. The device system is completely removed and disposed of in the recovery area

Briefly, an over-the-needle catheter introducer tool was used to create a para-incisional access point proximal to the incision site for introduction of the electrode lead. The needle was removed, leaving the catheter sheath in the surgical site. The electrode lead was fed through the catheter and placed proximal to the nerve repair site. The electrode lead was secured at the skin entry point using a Steri-Strip^TM^. To further secure the electrode lead, a small tension relief coil was created just proximal to the skin entry point and secured again using a Steri-Strip^TM^. Skin closure and dressing procedures were conducted as normal (Fig. 2). Post-operatively, the signal generator was connected to the electrode lead and adhered to the patient’s forearm using a hydrogel-based surface electrode that was attached to the bottom surface of the signal generator.

Where general anesthesia was applied, the patient was allowed to fully regain consciousness before initiating the therapy in the recovery room. Where local anesthetic was used, prior to initiating ES therapy, the anesthesia was allowed to dissipate in order to mitigate the effect of local anesthetic blocking the retrograde propagation of action potentials and thus the mechanism of action of ES therapy (Al-Majed et al. 2000). This was confirmed by waiting a minimum of 90 minutes from the last applied bolus of local anesthetic. Patients received test stimuli at 1 Hz and were asked to respond if the stimuli were perceived. If a patient could not feel the stimuli, the output of the stimulator was paused and the procedure repeated following a 15-minute wait time. Once the patient confirmed perception of the test stimuli, the therapy was initiated.

ES therapy comprised of the most studied stimulation paradigm of 20 Hz continuously for 1 hour (Barber et al. 2018; Gordon et al. 2010; Wong et al. 2015; Power et al. 2019) with stimulus levels (mA) adjusted such that effective stimulation could be confirmed by the patient and be comfortable for them.

The treating physician had the ability to set the electrode configuration during operation to either bipolar or monopolar stimulation.

After completion of ES therapy, Investigational PeriPulse^TM^ was removed by disconnecting the signal generator from the electrode lead and gently withdrawing the electrode lead from beneath the dressing, without the need for alteration. The device was disposed of in the recovery room prior to patient discharge from the hospital. There were no significant disruptions of the surgical wound or dressing. See Fig. 2 for a visual depiction of Investigational PeriPulse^TM^ implementation.

### Usability and patient tolerance questionnaires

Following each procedure, surgeons were asked to complete device usability and patient tolerance questionnaires. These questionnaires captured time for device implementation, application of the shapeable electrode lead, stimulation parameters, and patient responses during stimulation.

### Postoperative follow-up

At the first postoperative visit, typically 2–4 weeks following surgery, visual-analogue scale (VAS) pain scores were obtained. Participants also returned at 3- and 6-months post operation for sensory and disability assessments by a qualified hand therapist, further described below. These study endpoints parallel those studied by Wong et al. 2015.

### Visual-analogue pain score

Participants were asked to report their pain on a visual-analogue scale (VAS) out of 10, where 0 represents “no pain” and 10 represents “greatest pain ever experienced”.

### Semmes-Weinstein Monofilament Test (SWMT)

A SWMT 20 monofilament kit (Touch-Test® Sensory Evaluators, Remington Medical, Markham, ON, Canada) was used to assess the pressure detection threshold. Ascending “method of levels” was used to test in the sensory autonomous zone of the affected digital nerve. Force was applied until the monofilament was bent. The detection threshold was determined as the smallest fiber in which 75% correct identification was achieved out of 4 applied stimuli.

### Static 2-Point Discrimination (S2PD)

A static 2-Point discriminator (Touch-Test® 2 Point Discriminator, Remington Medical, Markham, ON, Canada) with pin distance 1–15 mm was used to assess spatial discrimination. Ascending “method of levels” was used to test in the sensory autonomous zone of the affected digital nerve. Force was applied just until skin blanching. The spatial discrimination result was determined as the smallest pin distance in which 75% correct identification was achieved out of 4 applied stimuli.

### Disability of the Arm, Shoulder, and Hand (DASH) questionnaire

Participants completed the disability module and any other applicable optional modules (e.g., work and/or sport) of the standardized Disability of the Arm, Shoulder, and Hand (DASH) questionnaire.

### Statistical analysis

Data are reported as mean ± SD, unless otherwise specified. Sensory data was analyzed using a repeated measures mixed-effects model for Gessier-Greenhouse correction. Multiple comparisons were done by Fisher’s LSD test with significance defined as *p* ≤ 0.05.

## Results

**Fig. 3:**
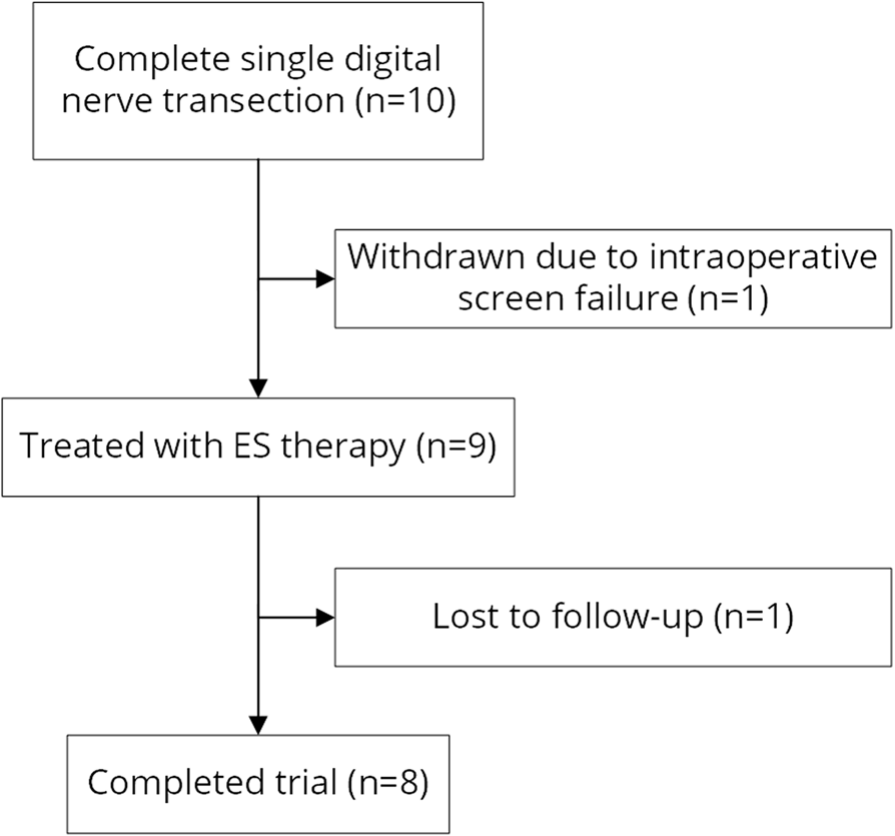
Clinical trial flow chart depicting the recruitment and follow-up of all study participants. One participant was withdrawn prior to ES therapy as they failed to meet the eligibility criteria of a fully transected nerve. 9 participants were treated with ES therapy using Investigational PeriPulse^TM^ and 8 participants completed the trial

Between April 2021 and June 2023, ten participants presenting with complete single digital nerve lacerations were enrolled at two sites in Hamilton, Ontario, Canada. Nerve injuries were sustained from sharp lacerations due to glass or knife injuries. One participant was withdrawn prior to receiving ES therapy as it was determined through surgical exploration that the nerve was not transected (neuropraxia). One participant was lost-to-follow-up after receiving ES therapy. A total of 8 participants completed the trial (Fig. 3 and Table 1).

**Table 1:** Patient Demographics

**Variable**	**Value**
Mean age (years)	38 (range 21–59)
Female (%)	40
Male (%)	60
Smoker (n)	1^*^
Dominant hand injured (n)	5
Concomitant tendon injury (n)	0
Time off work (n)	3
Digit injured (n)	
D1 (thumb)	0
D2 (index)	1
D3 (long)	3
D4 (ring)	1
D5 (little)	5

^*^Participant was withdrawn prior to treatment

All patients were included for safety analysis and no adverse events were reported that were deemed to be related to the study.

In the majority of cases, the bipolar electrode configuration was used (62.5%, surgeon preference due to ease of implementation and not having to connect a return electrode). There were no reported incidents of electrode lead migration which was determined by monitoring the stimulus intensity, patient responses, and patient movement. As the electrode lead was adhered on the forearm using Steri-Strip^TM^ and tightly secured underneath the dressing, it was unlikely to migrate during the therapy. All participants reported tolerable levels of stimulation and, importantly, no ES therapy was discontinued early (100% patient tolerance to stimulation). Stimulus levels were 2.6 ± 0.6 mA (range 2.1–3.5 mA). At the first postoperative visit, the average VAS pain score was 0.6 out of 10 (range 0–1.9 out of 10).

**Fig. 4:**
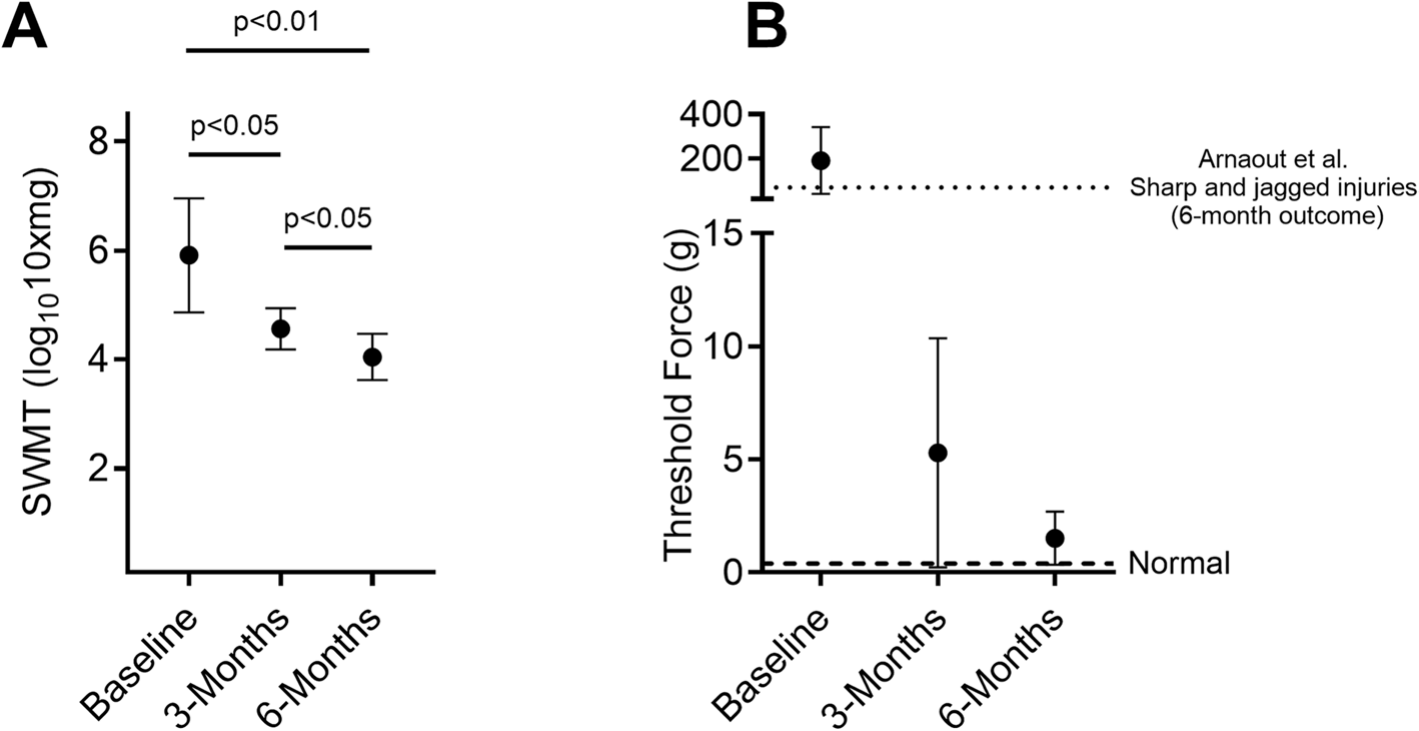
Mean and standard deviation of the Semmes Weinstein Monofilament Test (SWMT) pressure thresholds assessed at baseline, 3 months, and 6 months after digital nerve repair and ES therapy delivered using Investigational PeriPulse^TM^. **A** SWMT pressure thresholds at 3 and 6 months were significantly lower than scores at baseline (*p*< 0.05 and *p* < 0.01, respectively). Statistically significant improvement was also shown between 3 and 6 month scores (*p*< 0.05). Note, these values are the standard filament values which represent the logarithm of ten times the force in milligrams of each filament. **B** Pressure threshold scores represented in grams of force. Baseline thresholds represent patients that were feeling deep pressure only or were insensate. Dashed line represents the force an uninjured person would feel on the lateral or medial portion of the distal aspect of the digit (< 0.4 g). A historical comparator from the literature, Arnaout et. al shown in dotted line at mean value 68 g. This includes patients in the Arnaout study with both sharp or jagged lacerations

Sensory assessments were conducted at baseline, 3 months, and 6 months following surgery. The SWMT score at baseline was 5.91 ± 1.05 which corresponds to a clinical category of loss of protective sensation or deep pressure sensation only. The SWMT score at 3 and 6 months were 4.56 ± 0.38 and 4.05 ± 0.43, respectively. Statistically significant sensory recovery was observed at 3 and 6 months following digital nerve repair with ES therapy compared to baseline (Fig. 4A), with *p* ≤ 0.05 and *p* < 0.01, respectively. Sensory recovery after 6 months was also significantly better compared to that at 3 months (*p*≤ 0.05). While these values are represented in the log scale as corresponding to the test filament kit, true representation of the force thresholds are shown in Fig. 4B.

At 6 months post operation, 7 out of 8 (87.5%) patients who received ES therapy had improved clinical hand pressure thresholds (i.e., patients improved from deep pressure or loss of protective sensation at baseline to diminished protective sensation and diminished light touch), whereas only 1 patient (12% of total) remained with loss of protective sensation.

For participants treated with ES therapy, S2PD scores at baseline, 3 months, and 6 months were 12.8 ± 3.2, 8.5 ± 5.1, and 11.9 ± 3.6, respectively. There were no statistically significant differences.

**Fig. 5:**
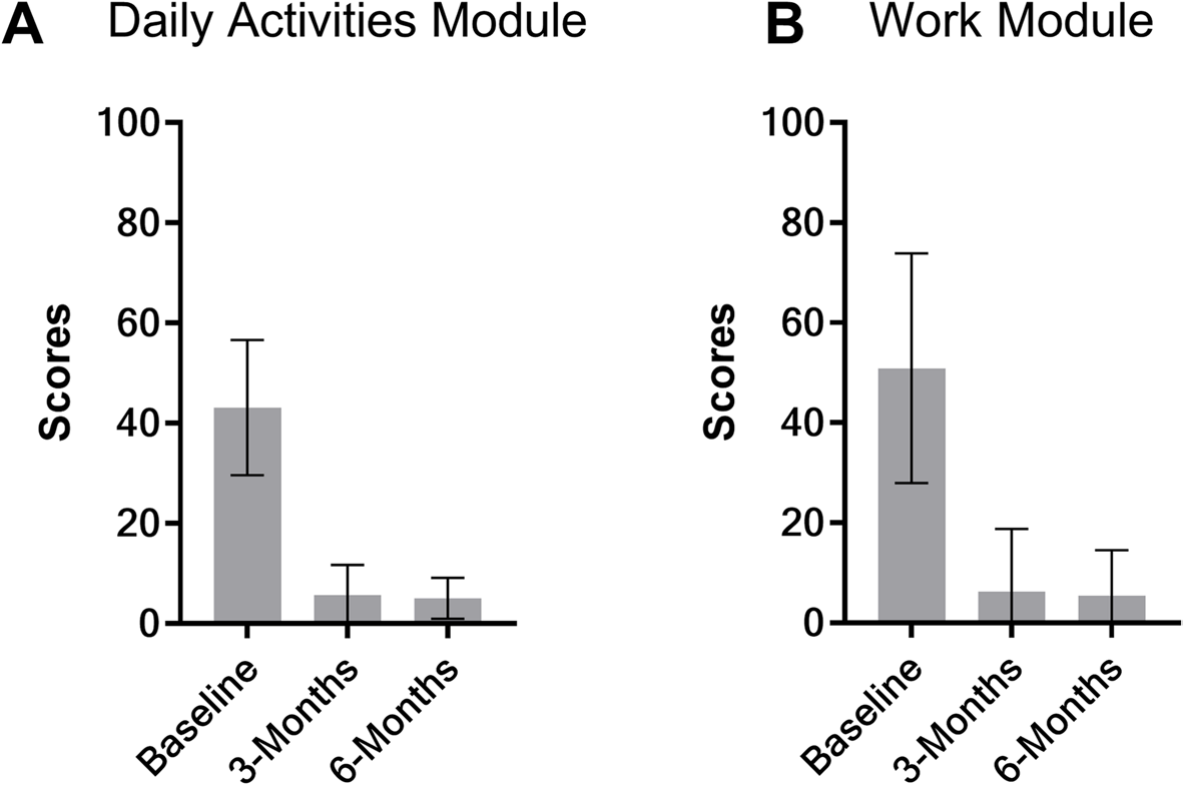
DASH scores at baseline, 3 months, and 6 months post operation and ES therapy for **A** daily activities questionnaire and **B** optional work module. In both modules, scores at 3 and 6 months were significantly lower than baseline (*p*< 0.05 and *p*< 0.01, respectively)

Patients reported a baseline DASH score of 43.15 ± 13.45. With ES therapy, patients reported significantly improved DASH scores of 5.73 ± 5.96 and 5.04 ± 4.11 at 3 and 6 months, respectively (Fig. 5A). Similarly, for work-related activities, patients reported a baseline score of 50.89 ± 22.94. With ES therapy, patients reported significantly improved DASH (work module) score of 6.25 ± 12.50 and 5.47 ± 9.11 at 3 and 6 months, respectively (Fig. 5B). There were no significant changes between 3 and 6 months.

## Discussion

Current approaches for delivering electrical stimulation therapy for injured peripheral nerves are not suitable for wide adoption and are largely unavailable to those outside academic centers who have ties to neuroscience labs where the traditionally used Grass SD9 stimulator is found. The use of 1-hour ES therapy has been promising and is based on robust preclinical and clinical research which has been extensively reviewed recently (Senger et al. 2024; Juckett et al. 2022; Wiebe and Borschel 2024; Liu and Fox 2024; Crabtree et al. 2024; Ni et al. 2023; Jin et al. 2023; Costello et al. 2023; Maeng et al. 2022; O’Brien et al. 2022; Willand et al. 2016). In the current study, ES therapy was delivered with the novel PeriPulse^TM^ neurostimulation system, which allows for perioperative implementation. The implementation of this device did not impact operating room time and was shown to be safe and highly usable as applied to digital nerve transections. Secondarily, sensory recovery was evaluated to document the extent of peripheral nerve regeneration was evaluated.

There is often a concern with patient’s feeling pain associated with the stimulation especially with stimulation of a transected nerve. However, in our study, patients did not complain of discomfort during stimulation. Stimulus levels were adjusted such that sensory capture was confirmed and were within the limits of patient tolerance (Wong et al. 2015). Patients did not report significant pain during stimulation and there was no request for termination of stimulation.

Perioperative ES therapy allows for personalized stimulus adjustment, whereas exclusive use intraoperatively would not, especially for repair or reconstruction of sensory nerves, where both sensory capture confirmation and determining the patient tolerance limit occur in the postoperative environment. Furthermore, perioperative implementation also enables the application of ES therapy in cases where short-acting local anesthesia is used. The electrode lead can be placed during the operation, which takes less than 5 minutes, and the stimulation may be started in the recovery room as soon as the effect of local anesthesia has dissipated. Perioperative implementation of ES therapy ensures that OR efficiency is not impacted.

Removal of the electrode was uneventful and there were no reports of pain. It was important to secure the electrode lead using an adhesive as it is possible to inadvertently move the electrode lead if not properly secured.

Bipolar stimulation was preferred over monopolar stimulation and presents numerous advantages. Application of monopolar stimulation requires a return electrode to be placed on the patient some distance away from the target nerve. Typical return electrodes could be a needle electrode (e.g., EMG type) which pose additional injury risks or surface electrodes. In the case of the investigational PeriPulse^TM^ system, the return was a hydrogel-based surface electrode which made application much safer. Nevertheless, bipolar stimulation provides a much more focused electrical field leading to a more targeted approach to treating an injured nerve. In this model, with stimulation taking place in the hand, the field is limited such that appropriate stimulation of only the injured nerve without affecting the adjacent digital nerve of the same digit, a bipolar configuration is more suitable.

Furthermore, patients reported very low pain scores at the first postoperative visit; however, the relationship between ES therapy and postoperative pain should be further studied in a larger patient population and in various clinical models.

We observed high variability in the S2PD results, which is not uncommon when using this test to assess outcomes (Lundborg and Rosen 2004; Bulut et al. 2016). Although S2PD is a widely accepted outcome measure for assessing sensory recovery, some authors have limited the use of this test due to its low reliability and high variability (Lundborg 2005; Lundborg and Rosen 2004; Bulut et al. 2016; Weinstein 1993; Bulut et al. 2018). We found at 6 months post operation, participants who received ES therapy did not have statistically significantly different S2PD scores from that described in the literature (Arnaout et al. 2014). Given that the autonomous zone for an isolated digital nerve is small, administering the S2PD test is a challenge for the assessor and hence higher variability is expected. Furthermore, it is difficult to standardize the applied pressure with most discriminator tools used to conduct S2PD. This further contributes to variability, especially when multiple assessors are involved (2005; Bulut et al. 2016; Bulut et al. 2018). In contrast, Semmes-Weinstein Monofilament Test inherently standardizes the amount of pressure applied and demonstrates lower variability and higher reliability than S2PD (Bulut et al. 2016; Weinstein 1993). Indeed, this is corroborated by our results, whereby we observe much lower variability within the SWMT data.

### Comparison with results in the literature

Although the vast majority of digital nerve studies in the literature (Dunlop et al. 2019) utilize S2PD due to the inherent simplicity and reduced time burden, SWMT, as outlined earlier, is considered a more reliable and specific test to measure nerve regeneration (Weinstein 1993). However, the number of studies for comparison is limited as other studies with digital nerve injuries did not necessarily evaluate SWMT at the same timepoints (3- and 6-months).

Two digital nerve studies with similar 6-month end points and outcome measures (Wong et al. 2015; Arnaout et al. 2014), one of which, Wong *et al.* utilized ES therapy (using a Grass SD9 stimulator) (Wong et al. 2015). Direct comparison however, was not possible as the Wong *et al.* study averaged data and normalized sensory values to the contralateral uninjured hand. This had lowered the variability and showed a significant difference between ES and sham groups.

Arnaout *et al.*’s paper evaluated the use of a conduit for protecting the repair site following direct repair of transected digital nerves (Arnaout et al. 2014). This study however, included patients with both sharp and irregular lacerations. Their sensory testing results were reported directly to allow comparison of SWMT results between the two studies. Interestingly, after 6 months, patients who received ES therapy in our study demonstrated reduced SWMT thresholds than that of the Arnaout et al. comparator group (primary repair alone). When represented in grams of force, as is typical in what a patient feels, pressure thresholds were approximately 45x lower with ES therapy than thresholds in the comparator group at 6 months post operation (1.52 ± 1.16 g vs 68.6 ± 126 g, Fig. 5B). While this includes patients with sharp and irregular injuries in the Arnaout study, isolating for only sharp lacerations still results in ES therapy having a superior result (1.52 ± 1.16 g vs 57.9 ± 120 g). Additionally, 15 out of 27 (55%) of Arnaout et al. patients who received primary repair alone, remained with “loss of protective sensation” and “deep pressure only” clinical thresholds (i.e., 55% of patients did not respond to surgery) which accounts for the large standard deviations seen in their data. Conversely, all patients that received ES in our study improved from baseline (i.e., 100% responder rate to surgery followed by ES therapy).

In the current study, perioperative ES therapy demonstrates improved sensory recovery in a Sunderland V injury and repair model. Patients who received ES therapy had better sensory recovery than at baseline (pre-repair) and were able to return to general daily and work-related activities within 3 months. This is clinically significant as patients with this nerve transection often experience slow recovery, up to 12–24 months for functional recovery (Goldie et al. 1992). Furthermore, after 6 months from surgery with ES therapy, a greater proportion of patients had clinically significant improved sensory recovery, than without ES therapy compared to a historical comparator group. Subjects in our study reported an improved quality of life over time, supporting the merit of ES treatment. These results implicate the profound beneficial impacts of perioperative 1-hour ES therapy, since without it, patients experience prolonged disability and loss of productivity, which have corresponding costs to society (Thorsén et al. 2012).

The DASH scores almost normalized by the end of the study, with most patients resuming almost all regular daily activities by 3 months after surgery. The work module DASH scores similarly were consistent with functional capacity to return to preinjury work. Our results are comparable to that which is described in the literature, wherein patients treated with ES therapy have a near normal DASH score by 6 months (our mean ± SD DASH score was 5.04 ± 4.11, which is similar to that described by (Wong et al. 2015) of 3.33 ± 1.21). 

A major limitation of our pilot study is a small sample size. Additionally, there was no sham treated or control group. However, the results of improved post-operative sensation from ES therapy treated patients remain promising as compared to historical data. The COVID- 19 pandemic contributed to the challenges of recruiting patients during hospital shutdowns.

## Conclusions

This pilot study demonstrated safety and usability of the novel ES device in digital nerve injury lacerations. In addition, we were able to collect data to determine effect size and confirm methodology for a large multicenter randomized controlled trial that is sufficiently powered and now underway (ClinicalTrials.gov Identifier: NCT05721261). This paper provides further evidence to support the well established 1-hour ES therapy paradigm (McLean and Verge et al. 2016, O’Brien et al. 2022, Padovano et al. 2022, Park et al. 2015) and introduces a novel device and method.

## Data Availability

Data is provided within the manuscript
