# Correction: Clinical evaluation of a novel disposable neurostimulator used to accelerate regeneration of injured peripheral nerves in the hand

**DOI:** 10.1186/s42234-025-00184-7

**Published:** 2025-09-01

**Authors:** Christopher J. Coroneos, Carolyn Levis, Michael P. Willand, Katelyn JW. So, James R. Bain

**Affiliations:** 1 Hamilton Health Sciences, 237 Barton Street East, Hamilton, Ontario L8L 2X2 Canada; 2 Division of Plastic Surgery, St. Joseph’s Healthcare Hamilton, 50 Charlton Avenue, Hamilton, Ontario L8S 4K1 Canada; 3 Division of Plastic Surgery, McMaster University, 1200 Main St W, Hamilton, Ontario L8N 3Z5 Canada; 4 Epineuron Technologies Inc., 1875 Buckhorn Gate Suite 602, Mississauga, Ontario L4W 5P1 Canada

**Correction:** ***Bioelectron Med*** **11, 9 (2025)**

10.1186/s42234-025-00171-y

Following publication of the original article (Coroneos et al. 2025), the authors reported an error in the Results section of the published article. The correct statement is provided below.

Incorrect:

At 6 months post operation, **5** out of 8 (87.5%) patients who received ES therapy had improved clinical hand pressure thresholds (i.e., patients improved from deep pressure or loss of protective sensation at baseline to diminished protective sensation and diminished light touch), whereas only 1 patient (12% of total) remained with loss of protective sensation.

Correct:

At 6 months post operation, **7** out of 8 (87.5%) patients who received ES therapy had improved clinical hand pressure thresholds (i.e., patients improved from deep pressure or loss of protective sensation at baseline to diminished protective sensation and diminished light touch), whereas only 1 patient (12% of total) remained with loss of protective sensation.

The original article (Coroneos et al. 2025) has been corrected.

## References

[CR1] Coroneos CJ, Levis C, Willand MP, et al. Clinical evaluation of a novel disposable neurostimulator used to accelerate regeneration of injured peripheral nerves in the hand. Bioelectron Med. 2025;11:9. 10.1186/s42234-025-00171-y. 40275339 10.1186/s42234-025-00171-y PMC12023366

